# Simultaneous measurement of 13 circulating vitamin D3 and D2 mono and dihydroxy metabolites using liquid chromatography mass spectrometry

**DOI:** 10.1515/cclm-2021-0441

**Published:** 2021-09-27

**Authors:** Carl Jenkinson, Reena Desai, Andrzej T. Slominski, Robert C. Tuckey, Martin Hewison, David J. Handelsman

**Affiliations:** Andrology, ANZAC Research Institute, University of Sydney, Sydney, NSW, Australia; Institute of Metabolism and Systems Research, University of Birmingham, Birmingham, UK; Department of Dermatology, University of Alabama at Birmingham, Pathology and Laboratory Medicine Service, VA Medical Center, Birmingham, AL, USA; School of Molecular Sciences, The University of Western Australia, Perth, WA, Australia

**Keywords:** liquid chromatography, mass spectrometry, metabolism, vitamin D

## Abstract

**Objectives:**

Clinical evaluation of vitamin D status is conventionally performed by measuring serum levels of a single vitamin D metabolite, 25-hydroxyvitamin D predominantly by immunoassay methodology. However, this neglects the complex metabolic pathways involved in vitamin D bioactivity, including two canonical forms D3 and D2, bioactive 1,25-dihydroxy metabolites and inactive 24-hydroxy and other metabolites.

**Methods:**

Liquid chromatography-tandem mass spectrometry (LC-MS/MS) can measure multiple analytes in a sample during a single run with high sensitivity and reference level specificity. We therefore aimed to develop and validate a LC-MS/MS method to measure simultaneously 13 circulating vitamin D metabolites and apply it to 103 human serum samples.

**Results:**

The LC-MS/MS method using a Cookson-type derivatization reagent phenyl-1,2,4-triazoline-3,5-dione (PTAD) quantifies 13 vitamin D metabolites, including mono and dihydroxy-metabolites, as well as CYP11A1-derived D3 and D2 metabolites in a single run. The lower limit of quantitation was 12.5 pg/mL for 1,25(OH)_2_D3 with accuracy verified by analysis of National Institute of Standards and Technology (NIST) 972a standards. Quantification of seven metabolites (25(OH)D3, 25(OH)D2, 3-epi-25(OH)D3, 20(OH)D3, 24,25(OH)_2_D3, 1,25(OH)_2_D3 and 1,20*S*(OH)_2_D3) was consistently achieved in human serum samples.

**Conclusions:**

This profiling method can provide new insight into circulating vitamin D metabolite pathways forming the basis for improved understanding of the role of vitamin D in health and disease.

## Introduction

Most research investigating the role of vitamin D in human health and disease has relied on the measurement of total 25-hydroxyvitamin D (25(OH)D) to assess vitamin D status [1]. The predominant platforms for 25(OH)D measurements originated with protein binding assays and are now mostly still performed by automated direct (non-extraction) immunoassays [2]. These are subject to suboptimal specificity due to cross-reaction with structurally related vitamin D metabolites. For example, cross-reactivity with 3-epi-25(OH)D3 and 24,25-dihydroxyvitamin D3 (24,25(OH)_2_D3), and underestimating the contribution of 25(OH)D2 levels to apparent total 25(OH)D [3] especially where vitamin D2 is taken as a nutritional supplement [2]. In addition, unextracted direct immunoassays have complex design making them subject to non-specific matrix and other artefacts [3]. It is therefore desirable to have specific analytical methods capable of measuring both D3 and D2 metabolites specifically, sensitively and accurately within a single run to gain a greater understanding of vitamin D metabolism and its role in health and disease.

The emergence of liquid chromatography-tandem mass spectrometry (LC-MS/MS) methods have improved the ability to accurately measure 25(OH)D and other vitamin D metabolites [3], [4], [5], however it is still the minority method used for analysis in clinical pathology laboratories. For example, among laboratories enrolled in major external quality control (QC) programs, fewer than 20% of laboratories in the Vitamin D External Quality Assessment Scheme (DEQAS) [6] and College of American Pathologists programs [7] report LC-MS/MS methods. Distinct advantages of using LC-MS/MS include their sensitivity, removal of non-specific matrix interferences [8], and separation of structurally related isoforms based on their distinctive mass to charge (*m/z*) fragmentation values [4]. Owing to these advantages LC-MS/MS using standardised materials, is the preferred analytical method according to expert panels [9].

Measuring low-abundance metabolites generated by hydroxylation of 25(OH)D is a challenge for LC-MS/MS method development. For example, to avoid overestimating 25(OH)D3 levels, it is necessary to separate the stereoisomer 3-epi-25(OH)D3 [10] which circulates at approximately 5–10% of 25(OH)D3 concentrations [11]. As the biological role of 3-epi-25(OH)D3 is still not well understood, further clinical research using specific LC-MS/MS methods are required especially where circulating concentrations are high as in infant and paediatric samples [11].

As 24,25(OH)_2_D3 is the major catabolite of 25(OH)D3 produced by the enzyme 24-hydroxylase (CYP24A1) [12] and in circulation at 10–15% of 25(OH)D3 concentrations [2, 13, 14], analytical methods have been established to investigate its clinical role [13], [14]. Measurements of 24,25(OH)_2_D3 have been used in studies of chronic kidney disease [15], and to determine mutations of CYP24A1 [16] along with idiopathic infantile hypercalcaemia [14] resulting from mutation in CYP24A1. The ratio of 25(OH)D3/24,25(OH)_2_D3 has also been proposed as an indicator of efficacy of vitamin D supplementation [17], [18]. A further trihydroxy metabolite 1,24,25-trihydroxyvitamin D3 (1,24,25(OH)_3_D3), formed from 24,25(OH)_2_D3 is reported in mouse serum but its concentration in humans is not known [19].

The main bioactive form of vitamin D is 1,25(OH)_2_D formed by C1α-hydroxylation (via the enzyme CYP27B1) of 25(OH)D. Its circulating levels being three orders of magnitude lower (picomolar) than 25(OH)D3 is a challenge to the accuracy of immunoassays [5] and LC-MS/MS methods. However, more sensitive high-end later generation LC-MS/MS instrumentation together with selective derivatization can achieve such sensitivity while preserving reference level specificity. Favourable derivatization is achieved with Cookson-type reagents such as 4-phenyl-1,2,4-triazoline-3,5-dione (PTAD), 4-[2-(6,7-dimethoxy-4-methyl-3,4-dihydroquinoxalinyl)ethyl]-1,2,4-triazoline-3,5-dione (DMEQ-TAD) and Amplifex Diene reagent to improve analyte ionization and detection limits [4], [5]. Such derivatizations can achieve the required sensitivity for measuring circulating 1,25(OH)_2_D3, and in some cases 1,25(OH)_2_D2 as well [20]. Circulating levels of 1,25(OH)_2_D2 and 1,25(OH)_2_D3 combined range from 14 to 98 pg/mL [20]. Analogous derivatization reactions have also been used for other mono and dihydroxy vitamin D metabolites incorporated into multi-metabolite LC-MS/MS profiling [10], [14].

More recently, an alternative pathway for vitamin D metabolism via the cholesterol side chain cleavage enzyme (CYP11A1) has been described. This features the enzymatic hydroxylation of vitamin D3 into two main monohydroxy metabolites – 20*S*(OH)D3 and 22(OH)D3 [21]. This process can occur in human placenta, skin cells and adrenal glands incubated *ex-vivo* [22]. These metabolites are further hydroxylated to several dihydroxy metabolites, including 1α,20*S*(OH)_2_D3, 20,22(OH)_2_D3, 20*S*,23*S*(OH)_2_D3 and 20*S*,24*R*(OH)_2_D3 [21] which have biological activity *in vitro* including antiproliferative, anti-inflammatory, anti-cancer and photoprotective activities as potential regulators of skin function [23], [24]. The major product, 20(OH)D3, has *in vivo* anti-fibrogenic [25] and anti-melanoma activities [26]. An additional source of 20(OH)D3 and 22(OH)D3 would be UVB-induced photo-transformation of 20(OH)7DHC and 22(OH)7DHC produced in the skin [27]. In addition, CYP11A1 can metabolized D2 to 20(OH)D2 and downstream metabolites under *in vitro* and *ex-vivo* conditions [28]. Few analytical methods are reported to measure circulating levels of CYP11A1 derived vitamin D metabolites with small studies reporting that serum 20*S*(OH)D3 and 22(OH)D3 are present in the bloodstream at low nanomolar concentrations [24, 29–31]. To our knowledge the biological significance of these vitamin D metabolites in human health and disease remains unclear with quantification of these metabolites not reported in clinical studies.

The complex metabolic pathway of vitamin D and the biological activities of metabolites beyond 25(OH)D3 highlight the importance of establishing multi-metabolite profiling methods. The improved insight into vitamin D metabolism and action can advance the understanding of vitamin D in health and disease phenotype and progression [4]. In this study we developed a sensitive and specific multi-metabolite LC-MS/MS profiling method for comprehensive analysis of vitamin D status in single serum samples. This method aimed to measure downstream metabolites of 25(OH)D3 including 3-epi-25(OH)D3, 24,25(OH)_2_D3, 1,25(OH)_2_D3, as well as CYP11A1 vitamin D3 metabolites. It also aimed to measure the counterpart for D2 metabolites 25(OH)D2, 3-epi-25(OH)D2 and 1,25(OH)_2_D2, distinguishing them from their D3 forms. Herein, we report development and validation of LC-MS/MS method, along with clinical application by measurement of circulating concentrations of seven vitamin D metabolites in 103 human serum samples.

## Materials and methods

### Chemicals

Reference vitamin D standards (Supelco brand) 25(OH)D3, 25(OH)D2, 3-epi-25(OH)D3, 3-epi-25(OH)D2, 24,25(OH)_2_D3, 1,25(OH)_2_D3, 1,25(OH)_2_D2, 25(OH)D3-d3, 25(OH)D2-d3, 3-epi-25(OH)D2-d3, 24,25(OH)_2_D3-d6 and 1,25(OH)_2_D3-d3, along with the derivatization reagent PTAD were purchased from Sigma Aldrich. The metabolite 1,24,25(OH)_3_D3 was purchased from BOC Sciences. 20(OH)D3, 22(OH)D3 and 20*,*22(OH)_2_D3 were synthesised enzymatically by the action of recombinant CYP11A1 on vitamin D3, as described before [31]. 20(OH)D3-d3 was similarly synthesised from CYP11A1 acting on deuterated vitamin D3 (6,19,19-d3) (Sigma Aldrich). 20*S,*24*R*(OH)_2_D3 was synthesised by the action of CYP24A1 on 20OHD3 [30] with C24 stereochemistry being assigned by comparison to chemically synthesized standards [32]. 1α*,*20*S*(OH)_2_D3 was synthesised by the action of CYP11A1 on 1α(OH)D3 [33]. LC-MS grade, water, isopropanol, acetonitrile and formic acid were purchased from Chem Supply. LC-MS grade methanol was purchased from Merck. Methyl tert-butyl ether (MTBE) was purchased from RCI Labscan Limited. A SecurityGuard ULTRA cartridge and holder for UHPLC Phenyl 2.1 mm ID columns was purchased from Phenomenex. A Waters ACQUITY BEH phenyl column (1.7 μ 2.1 × 75 mm) was purchased from Waters Corporation. SRM 972a Vitamin D Metabolites in Frozen Human Serum was purchase from the National Institute of Standards and Technology (NIST). Vitamin D depleted mass spectrometry (MS) certified serum was purchased from Golden West Biologicals (USA).

### Preparation of standard solutions

Vitamin D standards were purchased as stock solutions (5, 50, 100 μg/mL) in ethanol, apart from CYP11A1 metabolites that were sourced in powdered form. Stock solutions were diluted in methanol to form working solutions to prepare standard curve and QC samples by spiking known concentrations into vitamin D depleted serum. All standard solutions and QC samples were stored at −80 °C in amber vials until use. Internal standards (25(OH)D3-d3, 25(OH)D2-d3, 3-epi-25(OH)D3-d3, 1,25(OH)_2_D3-d3, 24,25(OH)_2_D3-d6) were purchased as stock solutions (5, 50, 100 μg/mL) and 20OHD3-d3 was sourced in powdered form. Internal standards were diluted to a working solution in methanol that was used to spike directly into samples.

### Sample collection and ethical approval

Serum samples were obtained from 103 randomly selected healthy male volunteers who participated in the T4DM study [34], a placebo-controlled study investigating whether pharmacological testosterone treatment ameliorated impaired glucose tolerance or reversed newly diagnosed type 2 diabetes. Briefly, 1,007 men aged 50–74 years, with a large waist circumference (≥95 cm), a screening serum testosterone concentration of ≤14·0 nmol/L but without pathological hypogonadism were randomized to treatment with injectable testosterone undecanoate (1,000 mg) or placebo for 2 years. Samples from both placebo and testosterone treated groups were analysed.

### Serum extraction

Vitamin D analytes were extracted from 300 µL of serum with the addition of 20 µL mixed internal standard solution in 1.5 mL microcentrifuge tubes. The concentration of internal standard compound present in samples was 25(OH)D3-d3 30 ng/mL, 25(OH)D2-d3 5 ng/mL, 3-epi-25(OH)D3 8 ng/mL, 20(OH)D3-d3 0.5 ng/mL, 24,25(OH)_2_D3 8 ng/mL, 1,25(OH)_2_D3 100 pg/mL. Samples were subjected to protein precipitated by the addition of 450 µL isopropanol/water (50/50 *v/v*) and vortexed at high speed for 10 min then left for a further 15 min at room temperature followed by centrifugation at 9,000 rpm for 5 min. The sample supernatant was transferred into glass tubes for liquid-liquid extraction performed as described [14] with modifications. The extraction was carried out by the addition of 1 mL hexane followed by vortexing samples for 30 s and the addition of 1 mL MTBE vortexed for a further 30 s. Samples were frozen at −20 °C for 2 h and the resulting organic layer was transferred and evaporated to dryness under nitrogen at 50 °C. The dry residue samples were derivatized by the addition of 0.125 mg/mL PTAD dissolved in acetonitrile, incubating for 2 h at room temperature in darkness. The reaction was quenched with the addition of 20 µL water and samples were dried under nitrogen and reconstituted in 75 µL water/methanol (50/50 *v/v*) was then transferred into the well of a 96-well microtitre plate.

### LC-MS/MS analysis

Analysis was performed on an SCIEX Exion LC system couple to an SCIEX 6500 QTRAP mass spectrometer, using electrospray ionization in positive mode. The multiple reaction monitoring (MRM) was obtained using settings for the various transitions optimized by infusing pure standard for each analyte into the mass spectrometer. Unit mass resolution was used in both mass-resolving quadruples Q1 and Q3. A single qualifier and another quantifier ion (QI) were optimized for each analyte. The optimized MRM transitions of each analyte are shown in Supplementary Material, Table S-1. The acquisition method was split into three periods during the sample run to quantitate groups of metabolites based on retention time; period 1 0–8.6 min, period 2 8.6–16 min, period 3 16–26.2 min. The MS instrument parameters are displayed in the Supplementary Material, Table S-2. A Waters UPLC BEH Phenyl (2.1 × 75 mm, 1.7 µm) column was used for liquid chromatography separation of metabolites. The column temperature was set to 40 °C and the flow rate was 0.300 mL/min with a mobile phase consisting of A:water 0.1% formic acid, B:Methanol 0.1% formic acid with the following mobile phase gradient; 0 min: 38%-A:62%B, 0.01–12 min: 35%-A:65%B, 12.01–22.4 min: 28%-A:72%B, 22.41–25 min: 28%-A:72%B, 25.01–26.5: 38%-A:62%B. It was necessary to have two gradient steps at 12.01–22.4 and 22.41–25 min to achieve 72% methanol mobile phase composition by 22.4 min, and maintain this until 25 min into the sample run. The overall run time was 26.5 min. A 35 µL sample injection volume was used and the autosampler temperature was set to 10 °C.

### Data analysis

Generation of calibration curves for data acquisition and processing was performed using Analyst 1.6.3 (AB SCIEX). Calibration curves were generated by plotting peak area ratios of each analyte over internal standard against the respective analyte concentration (fit: linear, weight: 1/x). Correlations between metabolites measured in healthy donor serum samples were determined using Pearson two-tailed correlation. Passing–Bablok regression and Bland–Altman plots were used for plotting regression and bias of vitamin D metabolite measurements against NIST 972a serum target concentrations and correlations were determined using Kendall’s tau-b correlation coefficient.

### Method validation

The method was validated to assess selectivity, accuracy, precision and matrix effects with determination of the lower limits of detection (LOD) and quantification (LOQ) of each analyte according to FDA guidelines for method validation of bioanalytical methods [35]. A MS certified vitamin D depleted serum was used as a matrix for preparation of validation QC samples by spiking the matrix with known concentrations of analytes. QC samples were extracted and analysed as described for unknown serum samples.

Selectivity of the method was determined by extracting and measurement of a sample of vitamin D depleted serum fortified with and without the addition of vitamin D standards. The MRM transitions for each analyte were monitored to ensure no interfering signals were observed at the expected retention times, or directly before or after each analyte signal. Selectivity was also assessed by monitoring the calculated response ratio of QI and confirmatory ions (QI/CI) of each analyte and the internal standards. The QI/CI ratio was monitored across calibration series and unknown samples. Any selectivity interference would be assumed if QI/CI ratio variation was greater than 30%.

Between-day accuracy as well as within and between-day precision were determined at three (low, medium and high) QC concentrations. Accuracy was calculated from the mean of six replicates measurements at each QC level and compared with the nominal analyte concentration. Precision was calculated from six replicates for each QC level (within-day) and six replicates for each QC level per day for three consecutive days (between-day). Accuracy of the method was further assessed for 25(OH)D3, 25(OH)D2, 3-epi-25(OH)D3 and 24,25(OH)_2_D3 by comparing the measured values with NIST certified reference (NIST 972a) levels for these analytes. These measurements were performed across 23 separate sample batches in which a NIST reference sample was included. A list of certified or reference concentrations for each NIST sample is shown in Supplementary Material, Table S-3. The limit of detection (LOD) and lowest limit of quantitation (LLOQ) were determined by the lowest concentration of each analyte that gave signal-to-noise ratio of at least 3 (LOD) and 10 (LLOQ) with a coefficient of variation (CV%) of <20% across six replicates.

The extraction recovery, matrix effects and process efficiency was assessed and calculated as previously described by Matuszewski et al. [36]. Extraction recovery was assessed by comparing the analyte area of extracted QC samples with the peak area of analytes spiked in a solution of water/methanol (50/50%) at the same concentrations as QC samples. To consider the effects of matrix and not losses occurring during the extraction procedure, vitamin D depleted serum was extracted and then spiked with analytes and internal standards in a solution of water/methanol (50/50%) at the same concentrations as QC samples, followed by sample analysis. The peak area for each analyte from these samples was compared with the peak area of a neat solution spiked in methanol/water (50/50).

A total of 103 human serum samples were measured to quantify vitamin D metabolites using the developed LC-MS/MS method.

## Results

### LC-MS/MS method optimization

**Figure 1:**
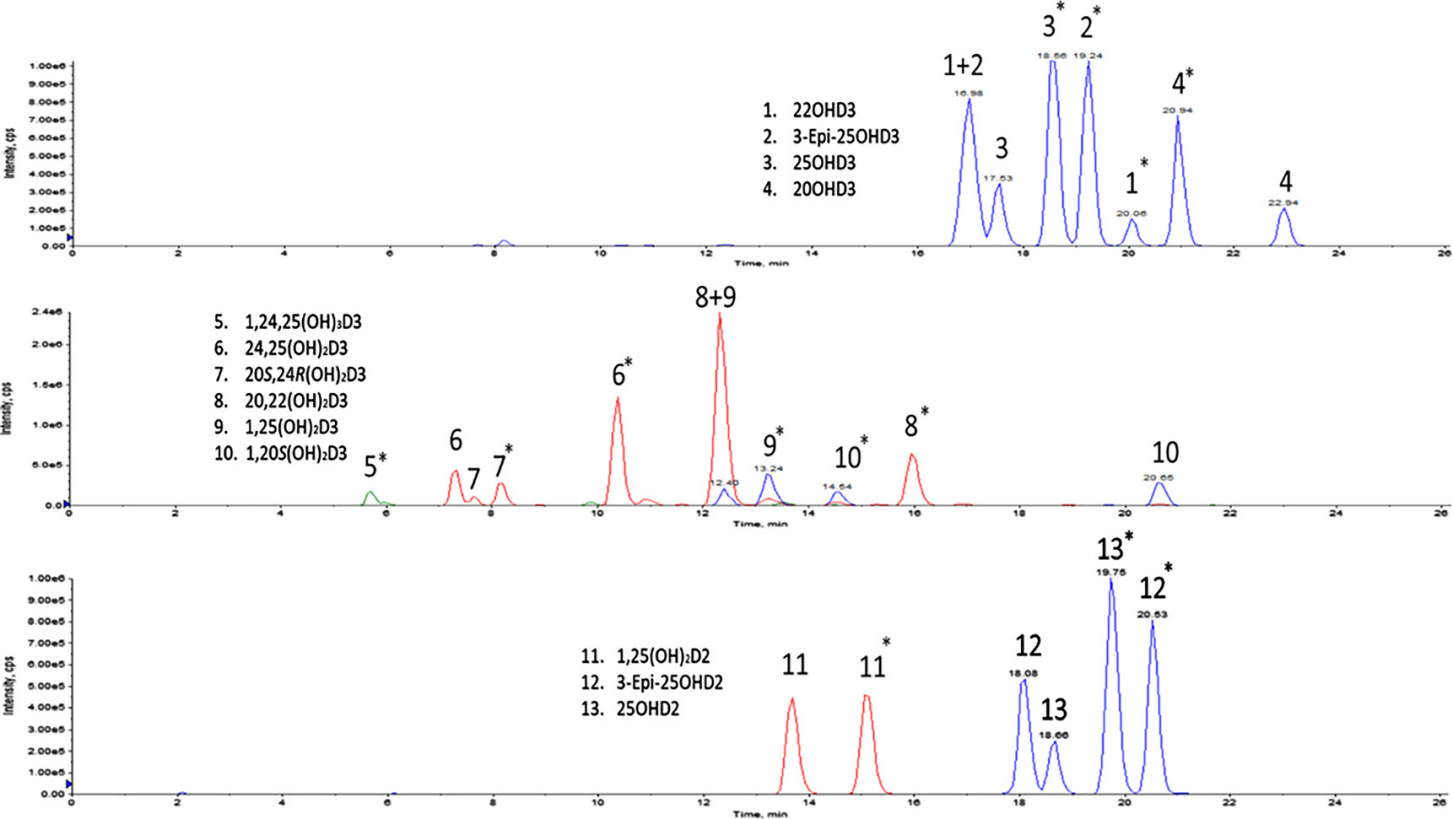
Chromatographic separation of 13 vitamin D metabolites in a single sample run from a vitamin D depleted serum spiked with known concentrations of vitamin D analytes, split into three panels. The concentration of 1,25(OH)_2_D3 extracted in the chromatogram was 2 ng/mL. A further chromatogram of 1,25(OH)_2_D3 at LLOQ (12.5 pg/mL) is displayed in Supplementary Figure S-3. *Indicates the analyte signal used for quantitation.

The addition of 0.125 mg/mL PTAD to samples was the optimum concentration achieving highest signal intensity for the derivatization of vitamin metabolites (Supplementary Material, Figure S-1). The optimized MRM transitions for each analyte following full scan and daughter scan analysis are displayed in Supplementary Material, Table S-1. An example of the fragmentation patterns of 1,25(OH)_2_D3 are shown in Supplementary Material, Figure S-2. The separation of 13 vitamin D metabolites is shown in Figure 1. A BEH Phenyl column enabled the separation of multiple groups of isomers from the 13 metabolites incorporated in the method. PTAD can bind to the *R* or *S* position on the vitamin D molecule producing two isomer pairs for each analyte [37]. When using the BEH Phenyl column these isomer pairs were eluted separately, producing two distinguishable eluting peaks for each analyte. As the ratio of each pair remains consistent across samples, we have selected one of each pair of signals for the quantitation of each analyte. The separation of metabolites by liquid chromatography included the important step of resolving groups of isomers that have similar or identical MRM transitions. This included separating the monohydroxy D3 metabolites (25(OH)D3, 3-epi-25(OH)D3, 20(OH)D3 and 22(OH)D3), dihydroxyvitamin D3 metabolites (24,25(OH)_2_D3, 20*S*,24*R*(OH)_2_D3, 20,22(OH)_2_D3, 1,25(OH)_2_D3, 1,20*S*(OH)_2_D3) and the monohydroxy D2 metabolites (25(OH)D2, 3-epi-25(OH)D2). This was achieved by applying a gradual increasing gradient of methanol mobile phase from 62 to 72% over a run time of 22 min.

### Method validation

#### Selectivity, linearity and LLOQ

The injection of an extracted vitamin D-depleted serum sample as a blank matrix confirmed no interfering peaks at the expected retention times of each analyte. The analysis of a blank matrix sample spiked with known concentrations of each vitamin D metabolite confirmed the presence of the following vitamin D metabolites at the anticipated retention times; 1,24,25(OH)_3_D3 [RT-5.6 min], 1α,25(OH)_2_D3 [RT-13.2 min], 1α,25(OH)_2_D2 [RT-15.0 min], 1a,20S(OH)_2_D3 [RT-14.5 min], 20S,24R(OH)_2_D3 [RT-8.2 min], 20,22(OH)_2_D3 [RT-12.3 min], 24,25(OH)_2_D3 [RT-10.4 min], 25(OH)D3 [RT-18.5 min], 25(OH)D2 [RT-19.7 min], 3-epi-25(OH)D3 [RT-19.2 min], 3-epi-25(OH)D2 [RT-20.5 min], 20(OH)D3 [RT-20.9 min] and 22(OH)D3 [RT-20.0 min]. There were no matrix interferences observed directly before or after the respective signals of each analyte.

The validation data showing the accuracy, precision recovery and matrix effects values are shown in Table 1. The within and between-day precision were all below 15%, and these values were consistent across analytes and QC levels. The accuracy values were within 15% of the expected QC concentration for all metabolites at low, medium and high QC levels. The LOD, LLOQ and correlation coefficients across a specified linear range for each analyte are displayed in Table 2, along with the accuracy and precision values at LLOQ. An example of the analyte signal of 1,25(OH)_2_D3 at LLOQ concentration is shown in Supplementary Material, Figure S-3. Further accuracy of the method was confirmed using NIST 972a serums and a comparison of LC-MS/MS measurements with NIST certified and target values is shown in Table 3.

**Table 1:** Accuracy, precision, recovery, matrix effects and process efficiency values of each vitamin D metabolite in the developed LC-MS/MS method.

Compound	Concentration, ng/mL	Accuracy and precision (CV)			Matrix effects and recovery		
		Within-day, % n=6	Between-day, % 6+6+6	Accuracy, % n=6	Matrix effects,% n=6	Recovery, % n=6	Process efficiency,% n=6
25(OH)D3	2.0	5.4	6.6	104.5	94.4	82.8	87.7
	20	1.4	2.0	99.1	90.4	74.4	82.3
	100	2.1	2.1	97.5	85.0	73.8	86.9
25(OH)D2	0.250	6.1	6.3	97.5	84.7	77.3	91.3
	3.0	2.7	4.3	94.4	85.1	72.2	84.9
	30	1.7	3.7	104.9	86.1	76.4	88.7
3-Epi-25(OH)D3	0.250	2.5	4.1	99.8	88.2	79.1	89.6
	3.0	3.8	4.6	98.0	96.0	76.2	79.4
	30	4.3	6.0	97.3	95.2	85.2	89.6
3-Epi-25(OH)D2	0.200	3.7	5.3	99.2	84.5	78.1	92.4
	1	3.1	4.2	91.9	94.1	64.7	68.7
	10	4.0	4.6	90.4	99.9	87.7	87.8
20(OH)D3	0.200	3.8	6.4	109.3	88.7	67.6	76.3
	1.0	3.8	4.3	104.9	90.9	77.8	85.6
	10	2.4	5.0	100.8	90.4	79.2	87.6
22(OH)D3	0.200	2.0	5.5	108.8	88.8	74.1	83.4
	2.50	3.7	3.9	101.0	82.4	69.9	84.8
	10	1.0	5.2	108.3	96.1	83.1	86.4
24,25(OH)_2_D3	0.250	6.9	7.2	94.7	86.8	75.3	86.7
	3.0	2.2	2.4	99.7	88.7	83.7	94.3
	30.0	3.1	2.3	102.0	97.6	85.9	88.0
1α,25(OH)_2_D3	20 pg/mL	6.6	5.4	96.8	90.8	90.5	99.6
	75 pg/mL	1.0	3.7	101.4	83.5	79.0	94.6
	250 pg/mL	1.2	2.9	102.0	88.4	87.9	99.4
1α,25(OH)_2_D2	10 pg/mL	11.5	8.1	93.9	96.9	93.8	96.8
	40 pg/mL	2.5	4.7	106.3	91.6	87.3	95.3
	150 pg/mL	3.2	5.2	112.3	90.0	89.0	98.9
20S,24R(OH)_2_D3	1.0 ng/mL	9.2	8.8	107.4	84.3	76.3	90.5
	4.0 ng/mL	0.9	1.7	106.3	90.7	82.9	91.4
	20.0 ng/mL	3.2	5.8	102.0	96.2	83.5	86.8
1a,20S(OH)_2_D3	100 pg/mL	9.2	6.1	92.1	92.8	90.8	97.8
	350 pg/mL	2.4	6.6	88.5	92.5	88.1	95.2
	2000 pg/mL	12.8	9.8	118.4	93.2	92.4	99.1
20,22(OH)_2_D3	50 pg/mL	1.8	5.4	102.7	82.7	65.8	79.5
	300 pg/mL	4.3	3.1	101.9	88.8	77.7	87.5
	600 pg/mL	5.9	3.6	105.2	86.5	78.9	91.2
20,22(OH)_2_L3	100 pg/mL	4.9	4.7	104.1	88.1	70.7	80.3
	500 pg/mL	1.0	1.8	100.0	93.3	82.6	88.5
	2,000 pg/mL	3.8	2.9	103.3	94.8	84.4	89.0
1,24,25(OH)_3_D3	50 pg/mL	6.9	5.1	96.3	81.4	75.0	92.1
	200 pg/mL	2.4	7.8	105.2	86.0	78.3	91.0
	2,000 pg/mL	5.1	9.5	103.9	88.5	83.8	94.7

The recoveries of the six monohydroxyvitamin D analysed ranged from 67.6 and 87.7% and that of the six dihydroxyvitamin D metabolite ranged from 65.8 and 93.8%. There were no significant signal enhancements or suppression across the vitamin D analytes based on matrix effects values. A minor ion suppression (15–20%) was observed for the following metabolites (QC concentration); 25(OH)D2 (0.250 ng/mL), 3-epi-25(OH)D2 (0.200 ng/mL), 22(OH)D3 (2.5 ng/mL), 1,25(OH)_2_D3 (75 pg/mL), 20*S*,24*R*(OH)_2_D3 (1 ng/mL), 20,22(OH)_2_D3 (50 pg/mL) and 1,24,25(OH)_3_D3 (50 pg/mL). Furthermore, the accuracy and precision values for these analytes was <15% at high, medium and low QC levels. Any ion suppression from these analytes is therefore unlikely to interfere with the accurate quantitation in samples. LC-MS/MS, liquid chromatography-tandem mass spectrometry; CV, coefficient of variation.

**Table 2:** LOD and LLOQ concentrations determined for vitamin D metabolites incorporated into the LC-MS/MS method.

Compound	Linear range, pg/mL	Correlation coefficient	LLOQ, pg/mL	LLOQ accuracy, %	LLOQ precision, CV%	LOD, pg/mL
25(OH)D3	176–380,000	0.998	20.0	99.3	10.0	10.0
25(OH)D2	29–60,000	0.997	29.3	109.0	6.5	14.7
3-Epi-25(OH)D3	25–26,000	0.998	25.4	107.1	16.9	12.7
3-Epi-25(OH)D2	16–8,000	0.988	31.0	104.5	8.3	16.0
20(OH)D3	31–32,000	0.998	63.0	119.3	13.3	32.3
22(OH)D3	31–32,000	0.998	63.0	95.8	13.0	32.3
24,25(OH)_2_D3	15–26,000	0.998	14.6	88.8	7.9	7.3
1α,25(OH)_2_D3	5–2,000	0.997	12.5	119.0	3.5	5.0
1α,25(OH)_2_D2	5–2,000	0.998	15.0	107.1	4.4	7.5
20S,24R(OH)_2_D3	100–2,000	0.997	100.0	106.8	10.4	75
1a,20S(OH)_2_D3	75–2,000	0.998	75.0	112.2	3.0	50
20,22(OH)_2_D3	15–2,000	0.997	15.0	97.1	10.9	5.0
1,24,25(OH)_3_D3	62.5–8,000	0.999	62.5	108.7	14.0	31.3

The CV% accuracy and precision values from six replicate QC samples at LLOQ are displayed. The correlation coefficient obtained at expected linear ranges is also displayed. LC-MS/MS, liquid chromatography-tandem mass spectrometry; CV, coefficient of variation; QC, quality control; LOD, limits of detection; LLOQ, lowest limit of quantitation.

**Table 3:** Regression, correlation and mean difference values for the comparison of LC-MS/MS measurements with certified and reference values for NIST972a samples. Measurement of NIST972a samples by LC-MS/MS was performed across 23 separate batches of sample analysis. The 95% limits of agreement of the mean differences are represented as the mean difference (1.96*SD).

Compound	Passing-Bablok			Bland–Altman
	Regression slope (95% CI)	Intercept (95% CI)	Correlation	Mean difference (1.96*SD)
25(OH)D3	1.04 (0.98, 1.1)	−1.04 (0.32, −2.24)	0.803	0.08% (2.10%)
25(OH)D2	1.09 (1.08, 1.12)	−0.06 (−0.04, −0.08)	0.761	2.42% (4.95%)
3-Epi-25(OH)D3	1.03 (0.99, 1.12)	−0.01 (−0.15, −0.48)	0.851	2.15% (3.85%)
24,25(OH)_2_D3	1.00 (0.98, 1.04)	−0.01 (−0.08, −0.03)	0.770	0.06% (1.46%)

Analysis of NIST 972a vitamin D metabolites in human serum samples confirmed the accuracy of measurements for 25(OH)D3, 25(OH)D2, 3-epi-25(OH)D3 and 24,25(OH)_2_D3. A comparison of LC-MS/MS measured concentrations for these analytes with the NIST certified and reference concentrations are shown by Passing–Bablok regression and Bland–Altman plots in Supplementary Figure S-4. Overall, these results indicate strong agreement between the measurements by LC-MS/MS from the developed method with the values of NIST. LC-MS/MS, liquid chromatography-tandem mass spectrometry; NIST, National Institute of Standards and Technology.

#### Application of the method

**Figure 2:**
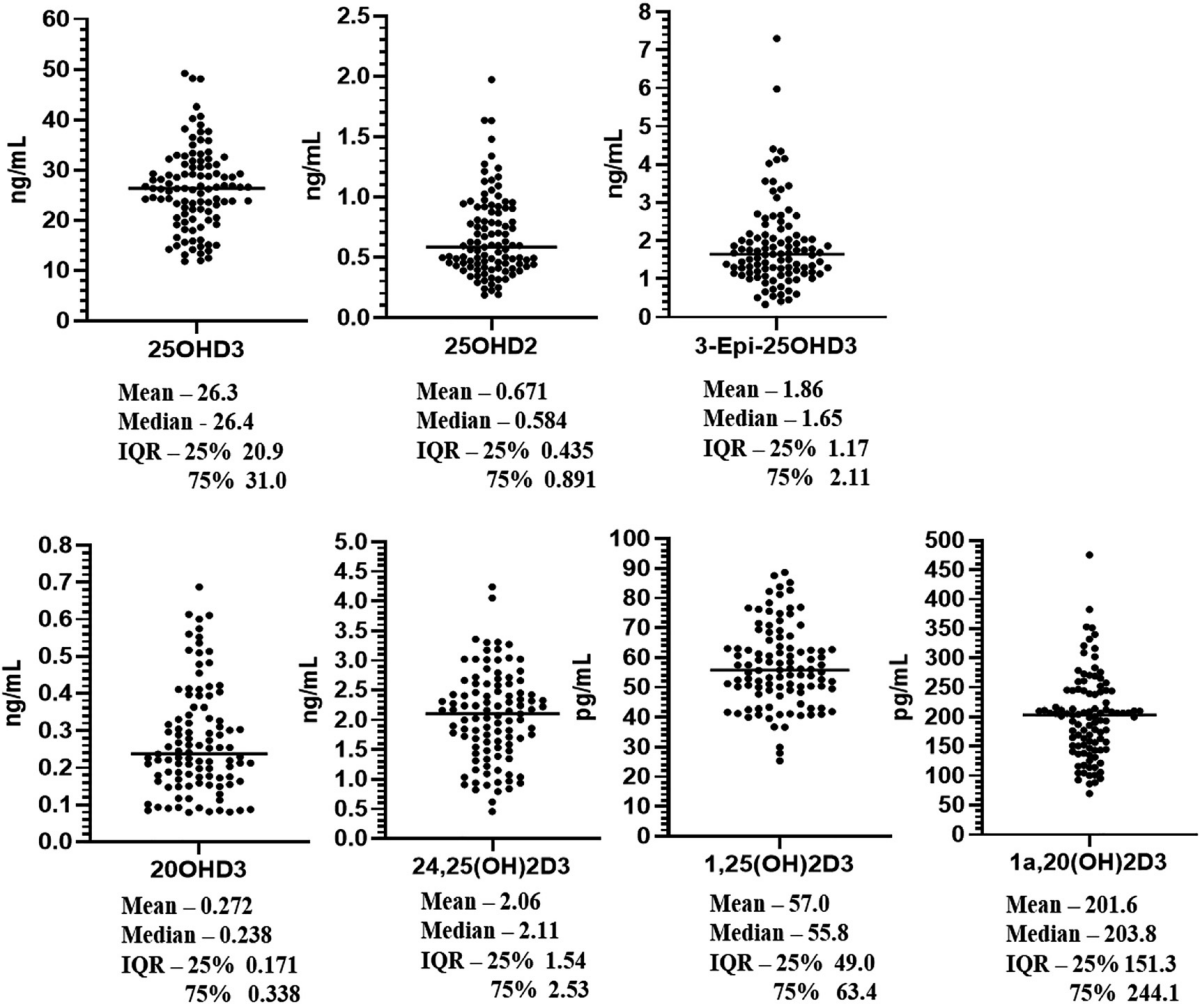
Concentrations of vitamin D metabolites measured in a cohort of healthy control donor serum samples.

The validated analytical method was used to measure vitamin D metabolites in 103 human serum samples. In this cohort the following seven vitamin D metabolites could be routinely quantified in samples – 25(OH)D3, 25(OH)D2, 3-epi-25(OH)D3, 20(OH)D3, 24,25(OH)_2_D3, 1,25(OH)_2_D3, 1a,20(OH)_2_D3. Figure 2 displays the individual measured concentrations of each analyte across the sample cohort. Correlations between pairs of vitamin D3 metabolites were observed in the sample cohort (Supplementary Material, Figure S-5) and the correlation coefficients for each pair of analytes are displayed in Table 4. This included strong associations between 25(OH)D3 and other D3 analytes, with the strongest 25(OH)D3 correlations occurring between 20(OH)D3, and 24,25(OH)_2_D3. The two CYP11A1 measured metabolites 20(OH)D3 and 1,20*S*(OH)_2_D3 also correlated with 25(OH)D3 measurements. A correlation between 25(OH)D3 and the biologically active 1,25(OH)_2_D3, was also observed. There was also a correlation between 1,25(OH)_2_D3 and 1,20*S*(OH)_2_D3 which may be explained by the involvement of CY27B1 in the 1α-hydroxylation both 25(OH)D3 and 20(OH)D3.

**Table 4:** Pearson two-tailed correlation coefficients (upper triangle) and their pairwise significance values (lower triangle) between vitamin D3 metabolites measured in 103 human serum samples.

	25(OH)D3	3-Epi-25(OH)D3	20(OH)D3	24,25(OH)_2_D3	1,25(OH)_2_D3	1,20*S*(OH)_2_D3
25(OH)D3		0.450	0.682	0.667	0.512	0.598
3-Epi-25(OH)D3	p<0.001		0.430	0.610	0.374	0.611
20(OH)D3	p<0.001	p<0.001		0.468	0.465	0.615
24,25(OH)_2_D3	p<0.001	p<0.001	p<0.001		0.388	0.688
1,25(OH)_2_D3	p<0.001	p<0.001	p<0.001	p<0.001		0.525
1,20*S*(OH)_2_D3	p<0.001	p<0.001	p<0.001	p<0.001	p<0.001	

Whilst our analytical method had incorporated a further six vitamin D analytes, the method did not achieve consistent measurements above the LLOQ in these samples studied for the following analytes; 3-epi-25(OH)D2, 22(OH)D3, 1α,25(OH)_2_D2, 20*S*,24*R*(OH)_2_D3, 20,22(OH)_2_D3 and 1,24,25(OH)_3_D3.

## Discussion

We describe a sensitive, specific LC-MS/MS method for the simultaneous profiling of 13 vitamin D metabolites. It has been applied to the analysis of human serum samples within which seven metabolites are consistently measurable in samples from healthy volunteers. This method enhances the breadth and accuracy of vitamin D analysis by resolving pairs of isomers by liquid chromatography with derivatization providing sufficiently sensitive detection limits to allow routine quantitation of seven metabolites in human serum, including the biologically active D3 metabolite 1,25(OH)_2_D3. Our wider profiling analysis of human serum samples is well aligned with previously reported circulating measurements of the same analytes in narrower profiles [10, 13, 14]. Measurements of 1,25(OH)_2_D3 were obtained across our measured sample cohort that align with other reported measurements by LC-MS/MS [20]. A correlation between 1,25(OH)_2_D3 and 25(OH)D3 was observed although not as strong when compared with that between 24,25(OH)_2_D3 and 25(OH)D3 indicating that this pair of analytes are not necessarily the best way to depict overall vitamin D status. However, in patients with glomerular disease lower 1,25(OH)_2_D3 was linked with decreased renal function, whereas 25(OH)D levels were not different [38] and lower levels of 1,25(OH)_2_D3 have been observed in patients with multiple sclerosis [39]. Higher 1,25(OH)_2_D3 concentrations have been associated with increased bone turnover and poorer bone health [40]. However, the optimal role of this bioactive vitamin D metabolite in bone or general health remains to be defined. The determined LLOQ of 1,24,25(OH)_3_D3 was 62.5 pg/mL which was above the circulating levels in our measured cohort. However the ability to quantify this analyte within this method will remain important in future studies to monitor whether there is an increased production of 1,24,25(OH)_3_D3 in populations, such as metabolism from high dosage of vitamin D3 supplementation or by increased metabolism of 1,25(OH)_2_D3.

Our analysis was also able to measure the CYP11A1 metabolites 20(OH)D3 (0.27 ± 0.34 ng/mL) and 1,20*S*(OH)_2_D3 (201.6 ± 71.3 pg/mL). However, we did not detect or quantify 22(OH)D3 in our human serum samples. In this cohort, 20(OH)D3 and 1,20S(OH)_2_D3 were measured at similar levels in the circulation, in contrast to a much wider difference in the more abundant circulating 25(OH)D3 and 1,25(OH)_2_D3. Studies *in vitro* using purified CYP27B1 have shown that 25(OH)D3 is a better substrate for 1α-hydroxylation by CYP27B1 [41] but it is possible that 1,20S(OH)_2_D3 has a longer serum half-life than 1,25(OH)_2_D3. The conversion of some vitamin D3 to 20(OH)D3 and its subsequent hydroxylation to the more active 1,20S(OH)_2_D3 could have important implications in vitamin D supplementation studies. The one previous study of these analytes using a LC-quadrupole time of flight MS method reported measurements of both 20(OH)D3 (1.15 ± 0.20 ng/mL) and 22(OH)D3 (2.38 ± 0.65 ng/mL) in 13 human serum samples [29], levels which are higher than the measurements in the present study. These differences in 20(OH)D3 and 22(OH)D3 concentrations may reflect the different study populations. Our sample cohort consisted of 103 males aged 50–74 years with impaired glucose tolerance, whereas the samples used by *Slominski* et al. [29] included a mainly female (10/13) but smaller population aged 25–61 years with no reported health conditions, collected at the end of summer. The serum samples used by *Slominski* et al. [29] also had higher 25(OH)D3 concentrations (33.6 ± 5.4 ng/mL) than in our measured sample cohort (26.3 ± 20.4 ng/mL). Circulating immune cells have context dependant expression of CYP27B1 and CYP11A1 which may affect localised levels of 25(OH)D3 and 20(OH)D3 and their 1α-hydroxylated counterparts within immune microenvironments [42]. Further studies are required to better understand the impact of CYP11A1 metabolism of vitamin D in different population groups and reference ranges for these metabolites. This includes probing different peripheral tissues expressing CYP11A1 such as skin and immune cells [42].

Our developed LC-MS/MS method incorporates the analysis of three vitamin D2 metabolites; 25(OH)D2, 3-epi-25(OH)D2 and 1,25(OH)_2_D2. Circulating levels of 25(OH)D2 in our sample cohort was low (1.62 ± 0.82 ng/mL) when compared to 25(OH)D3 measurements. We were unable to quantify 1,25(OH)_2_D2 or 3-epi-25(OH)D2 in any of our serum samples with the LLOQ concentrations of 15 and 31 pg/mL, respectively. A previous LC-MS/MS method reported by Shah et al. [43] measured circulating levels of 1,25(OH)_2_D2 (19 pmol/L) and 3-epi-25(OH)D2 (0.26 nmol/L) in 20 healthy subjects (10 male/10 female) with high 25(OH)D2 levels (19.7 nmol/L) when using a 1 mL plasma sample volume for analysis. We were unable to locate other previous reports of circulating 1,25(OH)_2_D2 or 3-epi-25(OH)D2 concentrations by LC-MS/MS. Monitoring these analytes will remain important in future studies for assessing vitamin D2 metabolism and potentially whether vitamin D2 was used for supplementation.

The aim of this method was to analyse vitamin D metabolism for the activation of vitamin D and the main inactive metabolites in this pathway, along with metabolites from the CYP11A1 pathway. Beyond these analytes there are further pathways for vitamin D that could provide future opportunities to advance this method. For example, a recent method established for 25,26(OH)_2_D3 analysis has confirmed circulating levels that could interfere with 24,25(OH)_2_D3 measurements [44]. Our method could also be expanded to investigate circulating levels of the 1β,25(OH)_2_D2 isomer of 1α,25(OH)_2_D2 [45], and metabolites further down the metabolic pathway. However this will be dependent on the extraction recovery and efficient ionization of these analytes following PTAD derivatization.

The present method enables an extensive multi-metabolite profiling approach for the analysis of vitamin D. This includes investigating different aspects of vitamin D metabolism including downstream metabolites of 25(OH)D3, 25(OH)D2 and CYP11A1 vitamin D3 metabolism. Our present study applied the developed LC-MS/MS method to set of clinical samples from 103 male volunteers aged between 50 and 74 years. Further studies utilizing this method within other population groups will enable a greater understanding of vitamin D metabolism at different age groups and whether there are any gender differences in metabolism as well as health status and disease progression. It must be noted that different vitamin D3 hydroxyderivatives have different selectivity for vitamin D receptor and other nuclear receptors including retinoic acid prophan receptors and arylhydrocarbon receptor [23], which should have important implications for their phenotypic activity. Furthermore, this combined assay could be used in veterinary medicine to assess the role of classical and non-classical (CP11A1-derived) metabolites as well as in research aimed at detection of different forms of vitamin D in natural products. This method will also be applicable in vitamin D supplementation trials that could inform whether oral ingestion of vitamin D has the same effects on vitamin D metabolites as endogenous synthesis from sunlight. The analytical method developed will therefore be an important tool for future analysis on the role of vitamin D in human health observational and clinical studies.

## Supplementary Material

Supplementary Material
