# Supplementary material for: Simultaneous measurement of 13 circulating vitamin D3 and D2 mono and dihydroxy metabolites using liquid chromatography mass spectrometry
Source: Clin Chem Lab Med. 2021 May 20;59(10):1642–52. doi: 10.1515/cclm-2021-0441 (PMC8419114; doi:10.1515/cclm-2021-0441)
Supplement: Supplementary file 1 — Supplementary Material [file j_cclm-2021-0441_suppl.docx]

**Table S-1.** Optimised MRM transitions for each analyte quantifier and confirmatory ions (QI/CI) and the optimised collision energies and dwell times. Quantifier ion is listed on the top row for each analyte.

| **Compound** | **m/z** | | **Molar mass** | **CE**  **(eV)** | **Dwell time (ms)** |
| --- | --- | --- | --- | --- | --- |
| 24,25-Dihydroxyvitamin D3  (24,25(OH)_2_D3) | 574.3 | 297.9  280.3 | 416.6 | 28.0  45.0 | 100  100 |
| 20S,24R-Dihydroxyvitamin D3  (20S,24R(OH)_2_D3) | 574.1 | 298.1  279.9 | 416.6 | 25.0  45.0 | 100  100 |
| 1,24,25-Trihydroxyvitamin D3  (1,24,25(OH)_3_D3) | 590.2 | 314.0  298.1 | 432.6 | 30.0  30.0 | 100  100 |
| 1α,20S-Dihydroxyvitamin D3  (1α,20S(OH)_2_D3) | 574.2 | 314.0  296.0 | 416.6 | 25.0  45.0 | 100  100 |
| 20,22-Dihydroxyvitamin D3  (20,22(OH)_2_D3) | 574.2 | 298.0  280.1 | 416.6 | 45.0  28.0 | 100  100 |
| 1α,25-Dihydroxyvitamin D3  (1α,25(OH)_2_D3) | 574.2 | 314.2  298.1 | 416.6 | 22.0  24.0 | 200  200 |
| 1α,25-Dihydroxyvitamin D2  (1α,25(OH)_2_D2) | 586.3 | 314.0  296.0 | 412.6 | 30.0  37.0 | 100  100 |
| 25-Hydroxyvitamin D3  (25(OH)D3) | 558.3 | 298.1  279.8 | 400.6 | 23.0  38.0 | 200  200 |
| 3-Epi-25-hydroxyvitamin D3  (3-Epi-25(OH)D3) | 558.3 | 297.9  280.1 | 400.6 | 23.0  35.0 | 100  100 |
| 20-Hydroxyvitamin D3  (20OHD3) | 558.3 | 297.8  279.8 | 400.6 | 24.0  33.0 | 100  100 |
| 22-Hydroxyvitamin D3  (22OHD3) | 558.3 | 298.1  280.0 | 400.6 | 25.0  36.0 | 100  100 |
| 25-Hydroxyvitamin D2  (25(OH)D2) | 570.3 | 297.9  279.9 | 412.6 | 27.0  37.0 | 100  100 |
| 3-Epi-25-hydroxyvitamin D2  (3-Epi-25(OH)D2) | 570.3 | 297.9  280.0 | 412.6 | 27.0  35.0 | 100  100 |
| **Internal Standards** |  |  |  |  |  |
| 24,25-Dihydroxyvitamin D3-d6  (24,25(OH)_2_D3-d6) | 580.3 | 298.1  279.9 | 422.6 | 28.0  45.0 | 100  100 |
| 1α,25-Dihydroxyvitamin D3-d6  (1α,25(OH)_2_D3-d6) | 577.3 | 301.1  317.2 | 419.6 | 25.0  22.0 | 200  200 |
| 25-Hydroxyvitamin D3-d3  (25(OH)D3-d3) | 561.3 | 301.1  283.0 | 403.6 | 22.0  35.0 | 200  200 |
| 3-Epi-25-hydroxyvitamin D3-d3  (3-Epi-25(OH)D3-d3) | 561.2 | 300.9  283.0 | 403.6 | 23.0  35.0 | 100  100 |
| 20-Hydroxyvitamin D3-d3  (20OHD3-d3) | 561.2 | 283.1  300.9 | 403.6 | 23.0  35.0 | 100  100 |
| 25-Hydroxyvitamin D2-d3  (25(OH)D2-d3) | 573.4 | 301.0  283.3 | 415.6 | 25.0  42.0 | 100  100 |

**Table S-2**. Mass spectrometry instrument parameters used for analyte detection.

| **Parameter** | **Value** |
| --- | --- |
| Curtain Gas | 20.0 PSI |
| Collision Gas | Medium |
| IonSpray Voltage | 4900 |
| Temperature | 316 ^o^C |
| Ion Source Gas 1 | 25.0 PSI |
| Ion Source Gas 2 | 70.0 PSI |
| Declustering Potential | 88.0 Volt |
| Entrance Potential | 10.0 V |
| Collision Energy | 45.0 V |
| Collision Cell Exit Potential (CXP) | 28.0 |

**Table S-3**. Certified (reference) values for 25OHD3, 25OHD2, 3-epi-25OHD3 and 24(OH)_2_D3 in NIST SRM 972a Levels 1-4.

| **Sample** | **ng/mL** |
| --- | --- |
| **Level 1** |  |
| 25OHD3  25OHD2  3-Epi-25OHD3  24,25(OH)_2_D3 | 28.8  0.54 (Reference value)  1.81  2.66 |
| **Level 2** |  |
| 25OHD3  25OHD2  3-Epi-25OHD3  24,25(OH)_2_D3 | 18.1  0.81  1.28  1.41 |
| **Level 3** |  |
| 25OHD3  25OHD2  3-Epi-25OHD3  24,25(OH)_2_D3 | 19.8  13.3  1.17 (Reference value)  1.62 |
| **Level 4** |  |
| 25OHD3  25OHD2  3-Epi-25OHD3  24,25(OH)_2_D3 | 29.4  0.55 (Reference value)  26.0  2.64 |

**Figure S-1**. Analyte areas of 25OHD3 and 1,25(OH)_2_D3 following the addition of PTAD at varying concentration ranges (n=2 for each PTAD concentration).


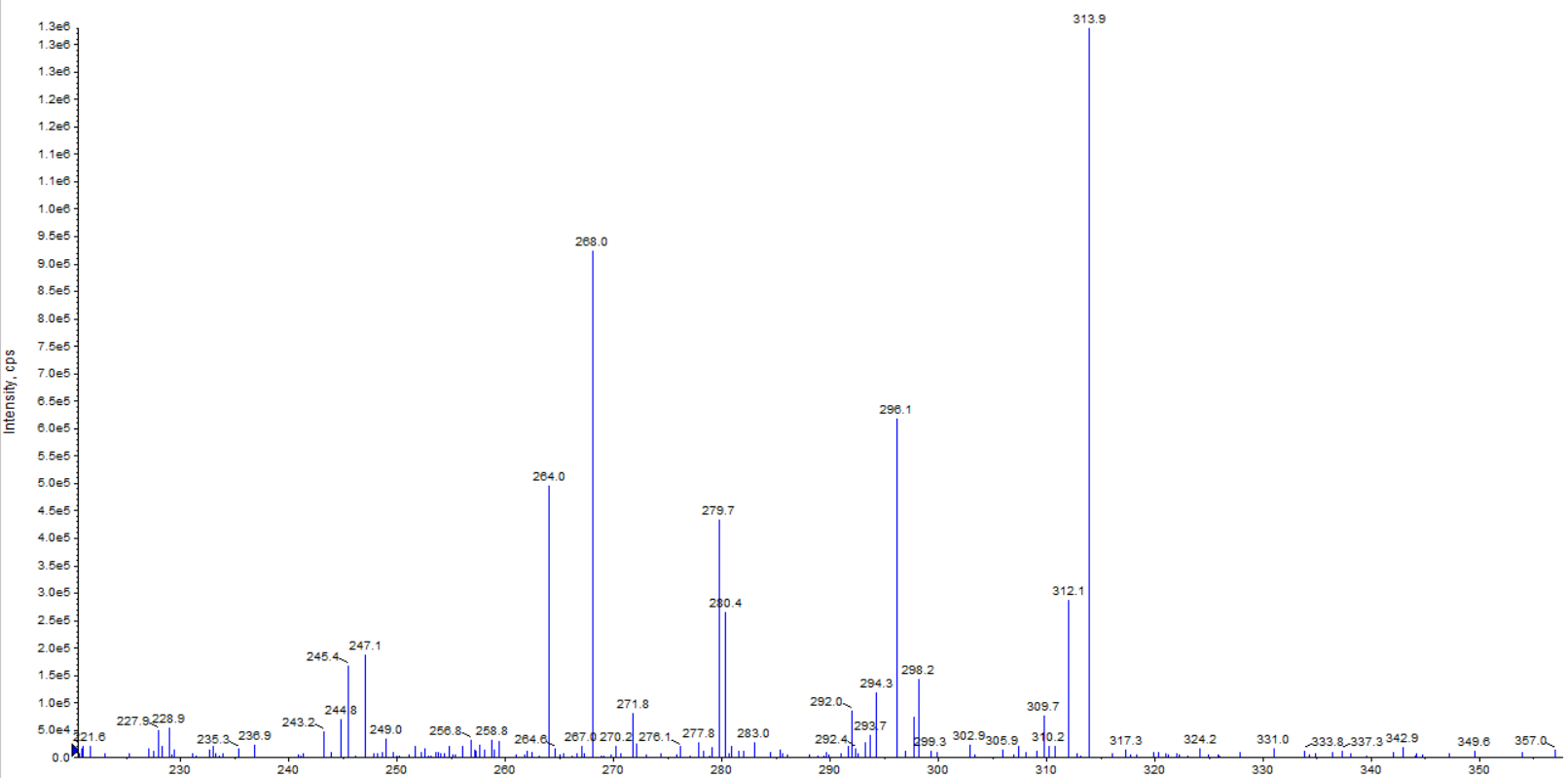


**Figure S-2**. Product ion spectrum of PTAD derivatized 1,25(OH)_2_D3. Total sum spectrum for 20-30 eV collision energy ramp.


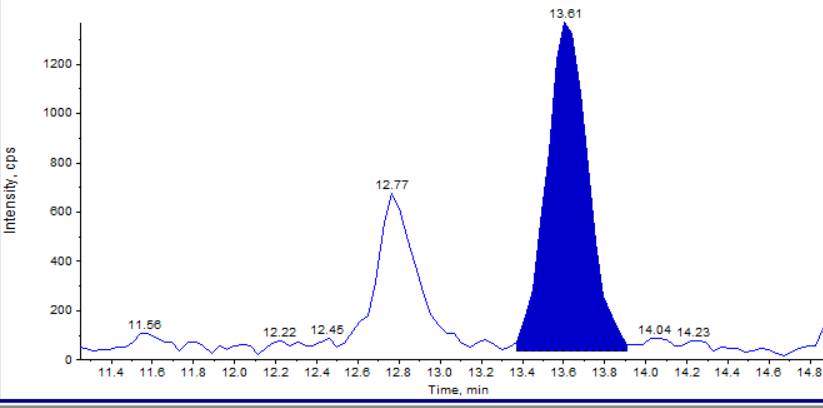


**Figure S-3**. Chromatogram of 1,25(OH)_2_D3 at LLOQ 12.5 pg/mL.


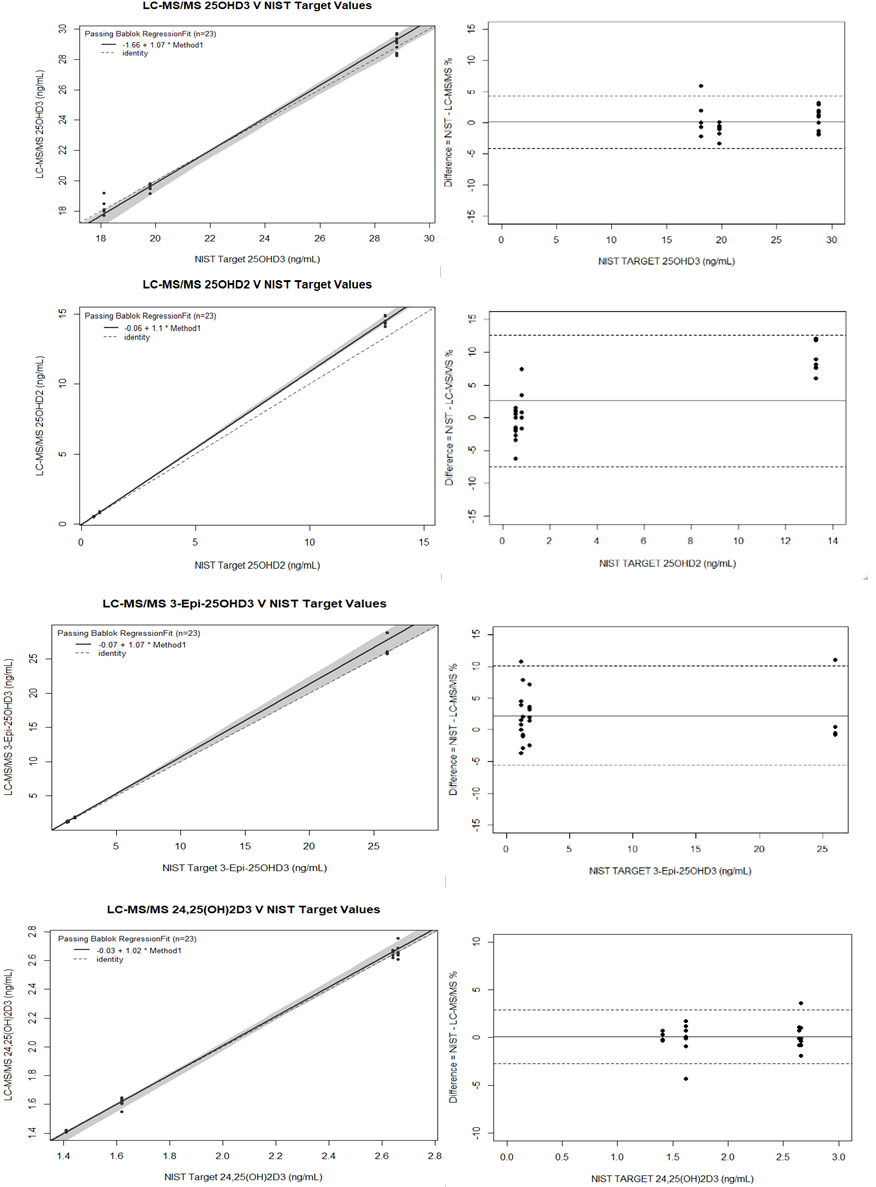


**Figure S-4**. Comparison of vitamin D metabolite measurements between the LC-MS/MS method with certified and reference values from NIST 972a serum samples. The sample measurements were obtained across the analysis of 23 separate sample batches. The solid line in the Passing-Bablok plots represents the regression line, the dotted line represents the line of identity. The solid line in the Bland-Altman plots represent the mean difference of measures against NIST values. The dotted lines represent the 1.96*SD values.

r-0.512

r-0.667

r-0.450

r-0.682

r-0.615


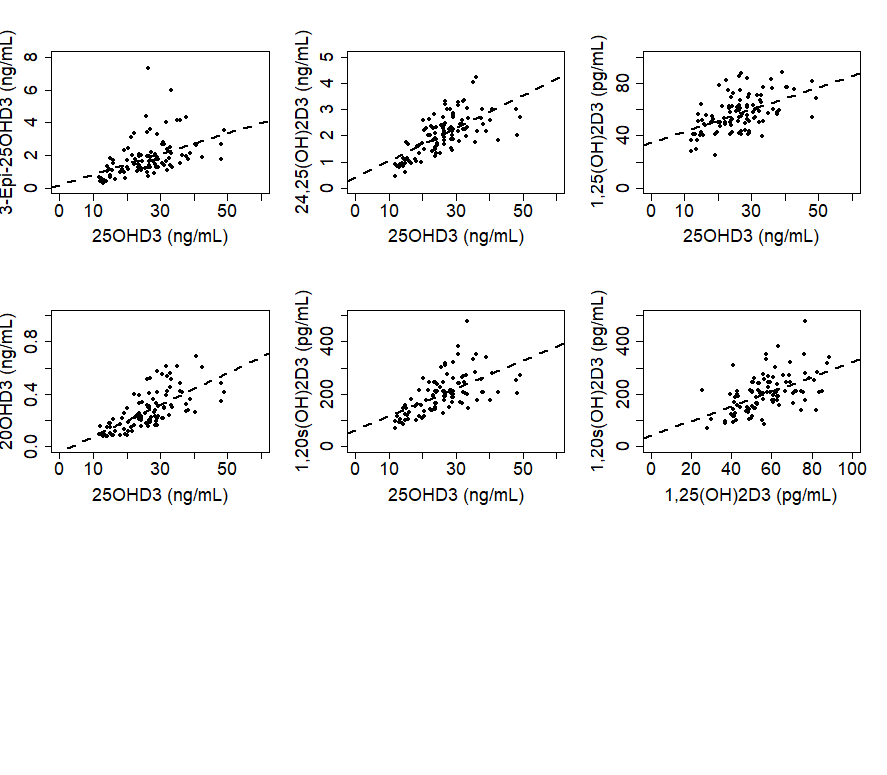

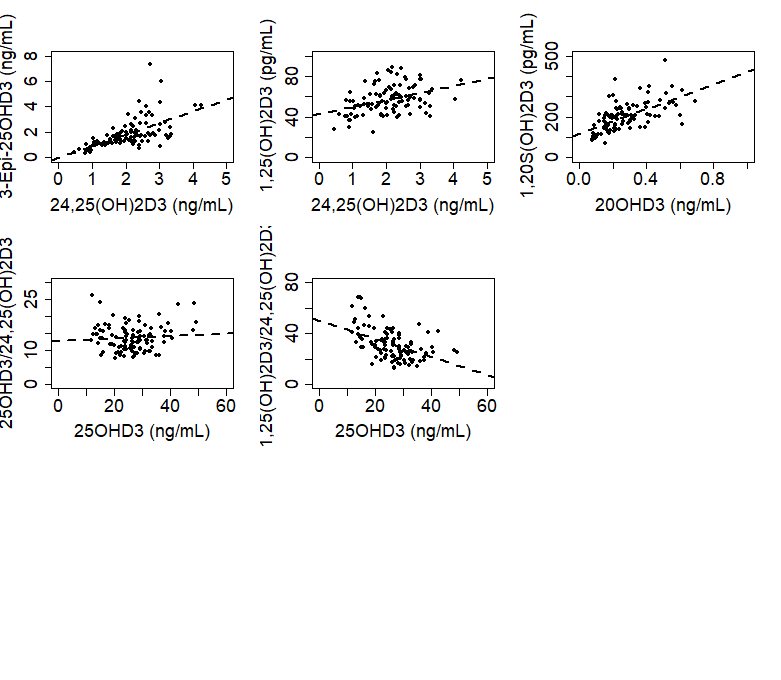


**Figure S-5.** Correlations between vitamin D3 metabolites in a cohort of healthy donor serums n-103.
